# The American Medical Association

**Published:** 1875-06

**Authors:** 


### Editorial.

---

THE AMERICAN MEDICAL ASSOCIATION, at its recent session in Louisville, seems to have retrieved its character, in part, as a representative body, in the estimation of some who have heretofore repeatedly visited it with sharp criticism. It is pleasant to read in the *Boston Medical and Surgical Journal:* "We have watched with great interest the progress of the meeting, and without as yet committing ourselves to a final opinion, we cannot but express our satisfaction with the general course of events. The leaders have appreciated their responsibility, knowing that to insure even the existence of the Association, it was necessary that this meeting should be a success. It is cheering to see in the list of those who visited Louisville the names of Gross, Atlee, and Wood, of Philadelphia; Wood, Flint, and Sayre, of New York; Toner and Woodward, of Washington; Eve, of Nashville; Jackson, of Danville; Jenks, of Detroit; Battey, of Georgia; Bowditch, Clarke, Morland, and Allen, of Massachusetts. Valuable contributions have been made to science, and the social attractions have been remarkably great. Strangers were received as friends, with the Southern hospitality for which Louisville is famous. Banquets, receptions and excursions occupied every leisure hour."

The *Medical Weekly* says: "The assembly has been a very large one; eminently harmonious and orderly; not a single incident occurring to mar the comfort and pleasure of the delegates. The officers elected are particularly acceptable." The *Medical Record* remarks: "The meeting just held in Louisville is the second one since the Judicial Council has had an existence, and already we begin to see the Association taking a stride towards respectability and usefulness, which it has not done for many years past. And the *Philadelphia Medical Times*, even, softens down somewhat, *e.g.:* "The meeting of the American Medical Association just closed appears to have been socially a very successful one, there having been a large number of delegates in attendance, and a universal spirit of fraternity and good feeling having prevailed. The presence of a large delegation from Boston was a novel and praiseworthy event. This delegation, we happen to know, went home in a state of great satisfaction with their trip; so that it is to be expected that the effort of our Eastern brethren will not be a spasmodic one, but that hereafter New England will always be well represented." Moreover, its authorized reporter ventures to say: "I believe it is generally conceded that the men are more than usually representative. Boston is especially well represented by such men as Drs. Bowditch and Clark, Dr. Bixby, an old partner of Dr. Storer, and Dr. Chadwick, the secretary of the District Medical Society; New York has some men of prominence, such as Flint, Sims, Sayre, and James R. Wood; and Philadelphia has a small delegation headed by Gross and Atlee. Probably to the superior character of the delegates are owing the grave decorum and general good feeling which have characterized the deliberations of the Association."

Having for the first time been privileged to look upon this national assemblage of our profession, we confess to a certain degree of deferential respect for the body. We were permitted to meet in friendly converse a very goodly number of delegates whom we willingly accept as representatives of the great American profession; a number by whose teachings we have gladly profited in the past, and many more at whose feet we would gratefully sit and learn lessons of golden wisdom in the future.

In a social point of view, we shall not soon forget the personal intercourse with men of sterling worth and solid attainments we there enjoyed. Many private courtesies, generously extended in an unostentatious way, struck a responsive cord in the heart which will long continue to vibrate.

The public receptions were numerous, brilliant and most enjoyable. Everywhere we marked, with pleasure, the absence of wine, and were not able to perceive that it detracted at all from the rational enjoyment of every guest. In the hospitable drawing rooms of Dr. D. W. Yandell we were greeted by a truly resplendent galaxy of female beauty, whose charm is not to be weighed in the balance with the punch-bowl and the too seductive effervesence of champagne.

For the proceedings of the body we must refer our readers to the copious extracts we publish elsewhere from exchanges, for we were too much occupied in private associations and enjoyments to feel any disposition to play the role of a reporter.

